# The Impact of Combining CIRS-G and Clinical Frailty Score on One-Month Mortality in Acute Coronary Syndrome

**DOI:** 10.3390/healthcare13222864

**Published:** 2025-11-11

**Authors:** Ahmet Yılmaz, Enes Çon

**Affiliations:** Department of Cardiology, Faculty of Medicine, Karamanoğlu Mehmetbey University, Karaman 70200, Turkey; enes_con@hotmail.com

**Keywords:** acute coronary syndrome, elderly, frailty, clinical frailty score, cumulative illness rating scale for geriatrics (CIRS-G), GRACE score

## Abstract

**Background/Objectives**: Acute coronary syndrome (ACS) remains a leading cause of short-term mortality, particularly in elderly patients with multimorbidity and frailty. Conventional models such as the GRACE score provide robust prognostication but do not incorporate comorbidity or frailty burden. This study investigated the prognostic value of combining the Cumulative Illness Rating Scale for Geriatrics (CIRS-G) and Clinical Frailty Score (CFS) with GRACE in predicting one-month mortality in older ACS patients. **Methods**: A single-center, retrospective cohort study was conducted including 90 patients aged ≥65 years admitted with ACS. Demographic, clinical, echocardiographic, and laboratory data were collected. CIRS-G, CFS, and GRACE scores were calculated at admission. The primary endpoint was one-month all-cause mortality. Statistical analyses included group comparisons, correlation tests, logistic regression, and ROC curve analysis. **Results**: The mean age was 74.8 ± 6.6 years, and 73.3% were male. At one month, mortality was 8.9% (n = 8). Non-survivors had significantly higher CIRS-G (median 18.5 vs. 14.0, *p* = 0.006), CFS (6.0 vs. 4.0, *p* = 0.008), and GRACE scores (183 vs. 122, *p* < 0.001), and lower ejection fraction (32.5 vs. 50.0, *p* < 0.001) compared with survivors. Logistic regression identified GRACE as the only independent predictor of mortality (OR = 1.081 per 10-point increase, *p* = 0.044). ROC analysis showed GRACE had the highest discriminative performance (AUC = 0.919), while CIRS-G (AUC = 0.796) and CFS (AUC = 0.777) also demonstrated significant predictive value. The combined CIRS-G + CFS model provided comparable discrimination (AUC = 0.785; sensitivity 75%, specificity 87%). **Conclusions**: GRACE remains the strongest independent predictor of one-month mortality in elderly ACS patients; however, comorbidity and frailty scores also contribute meaningful prognostic information. Integrating these geriatric assessments with traditional risk models may improve individualized risk stratification and management.

## 1. Introduction

Acute coronary syndrome (ACS) remains a major cause of morbidity and mortality worldwide, with substantial variations in its epidemiology, clinical presentation, and outcomes across populations and over time [1,2,3]. Advances in diagnostic tools, risk stratification models, and therapeutic strategies have improved survival rates, yet short-term mortality, particularly within the first month after an ACS event, continues to represent a significant clinical challenge [1,4]. Global data highlight the persistent burden of ACS in both developed and developing countries, with increasing recognition of heterogeneity in risk profiles and outcomes [5,6]. Traditional risk scores such as the GRACE score are widely used for mortality prediction and clinical decision-making, but they predominantly emphasize biomedical and hemodynamic parameters [2].

In recent years, there has been growing interest in the role of comorbidity burden and frailty in determining outcomes among older ACS patients. Epidemiological studies underline that multimorbidity and systemic vulnerability, beyond classical cardiovascular risk factors, significantly influence prognosis [3,5]. While the cumulative burden of illness has been captured by indices such as the Cumulative Illness Rating Scale for Geriatrics (CIRS-G), and frailty has been measured by scales such as the Clinical Frailty Score (CFS), the integration of these parameters into ACS risk prediction remains limited. Considering that older adults constitute a large proportion of ACS admissions and that frailty has been linked to worse in-hospital and post-discharge outcomes, combining comorbidity and frailty assessments may enhance prognostic accuracy beyond conventional models [1,2,6]. This approach could provide a more comprehensive understanding of early mortality risk and inform individualized management strategies.

This study evaluates whether comorbidity burden (CIRS-G) and frailty (CFS) provide incremental prognostic value beyond the GRACE score for predicting 1-month mortality in older patients with ACS, and explores their relationships with ICU admission, length of stay, and left ventricular ejection fraction. We hypothesize that integrating comorbidity and frailty assessments (CIRS-G and CFS) with the GRACE score will provide a more comprehensive prognostic evaluation and may enhance short-term mortality prediction in elderly ACS patients.

## 2. Materials and Methods

### 2.1. Study Design and Setting

This was a single-center, retrospective, observational cohort study conducted at the Department of Cardiology, Karamanoğlu Mehmetbey University Faculty of Medicine (Karaman Training and Research Hospital). In total, 90 consecutive patients aged ≥65 years who presented with acute coronary syndrome (ACS) and met the eligibility criteria were identified through the hospital electronic medical records and archived case files.

### 2.2. Participants (Eligibility Criteria)

Inclusion criteria: (i) age ≥ 65 years; (ii) index diagnosis of STEMI, NSTEMI, or unstable angina (USAP); (iii) calculable Cumulative Illness Rating Scale for Geriatrics (CIRS-G), Clinical Frailty Score (CFS), and GRACE score at admission; and (iv) availability of at least 1-month clinical follow-up.

Exclusion criteria: (i) known terminal malignancy; (ii) presentations due to trauma or non-cardiac causes; (iii) incomplete data or unavailable 1-month follow-up; and (iv) no coronary angiography/intervention performed during index admission. The final cohort comprised 90 consecutive eligible patients.

A minimum sample size of 82 patients was targeted based on a priori power analysis performed using G*Power software version 3.1.9.7 (two-sided α = 0.05, power = 80%) and calculated with IBM SPSS Statistics version 25.0.

Data sources and variables

Demographic characteristics (age, sex), cardiovascular risk factors and comorbidities (e.g., hypertension, diabetes mellitus, COPD, chronic kidney disease, heart failure, prior stroke, dementia), smoking status, index ACS type (STEMI/NSTEMI/USAP), echocardiographic left ventricular ejection fraction (EF, %), and in-hospital course (ICU admission, length of hospital stay) were abstracted from the hospital information system. Electrocardiograms, laboratory parameters (e.g., cardiac biomarkers [troponin, CK-MB, NT-proBNP], renal function tests [serum creatinine, estimated GFR], inflammatory markers [CRP, WBC], complete blood count and routine biochemistry), and coronary angiography reports were reviewed retrospectively.

Scores and definitions

- CIRS-G: Quantified comorbidity burden across organ systems according to standard scoring.
  - CIRS-G Index → Represents the average severity across all systems (numerical mean).
  - CIRS-G Grade → Each organ system is graded on a scale from 0 to 4.
  - CIRS-G score Grade 3–4 system number → Total number of organ systems with severe or very severe disease.
- Clinical Frailty Score (CFS): Assessed functional reserve and frailty status at presentation from documented clinical evaluations.
- GRACE score: Calculated per established methodology to estimate post-ACS mortality risk.

All scores reflected the patient’s status at index admission.

The primary outcome was 1-month all-cause mortality following the index ACS admission. Secondary outcomes included ICU admission, length of stay (days), and left ventricular ejection fraction (EF, %).

The data were de-identified prior to analysis and stored on secure, access-restricted drives. Records were screened against inclusion/exclusion criteria; cases with missing essential variables or incomplete 1-month follow-up were excluded, as prespecified.

The study protocol was reviewed and approved by the Karamanoğlu Mehmetbey University Faculty of Medicine Local Ethics Committee for Scientific Medical Research (approval date: 5 March 2025, approval number: 17-2025/12). The study was designed and conducted in accordance with the Declaration of Helsinki and Good Clinical Practice principles. As a retrospective analysis of existing records, no direct patient contact occurred.

### 2.3. Statistical Analysis

The statistical analysis of the data obtained in the study was performed using the SPSS 25.0 software (IBM Corp., Armonk, NY, USA). Continuous variables were examined through Q-Q plots to assess normality and supported with skewness and kurtosis criteria. Data were presented as mean ± standard deviation, median (Q1–Q3), minimum, and maximum. Categorical variables were expressed as frequency and percentage (%).

For group comparisons, the Independent Samples *t*-test and one-way analysis of variance (ANOVA) were used for normally distributed variables; for variables with significant differences in ANOVA, Bonferroni post hoc test was applied for pairwise comparisons. For non-normally distributed variables, the Mann–Whitney U test was employed. Comparisons of categorical variables were made using the Chi-square test. Relationships between variables were assessed with Pearson correlation analysis and, depending on the distribution, Spearman’s rho test. Interpretation of correlation coefficients was as follows: (Weak = 0.01–0.49; Moderate = 0.50–0.69; High = 0.70–1.00).

Binary logistic regression analysis was conducted to determine factors influencing survival. In this analysis, age, mode of presentation, CIRS-G score, CIRS-G index, Clinical Frailty Score, and GRACE score were included in the model. In addition, a separate logistic regression model was constructed to create a combined parameter, where only the CIRS-G score and Clinical Frailty Score were evaluated together to calculate the predicted probability. This probability value was defined as “CIRS-G + Frailty combination” and compared with the GRACE score and other parameters within the scope of ROC analysis.

ROC curve analysis was performed to examine the prognostic values of the variables; the area under the curve (AUC), sensitivity, specificity, and Youden index values were calculated. A *p*-value < 0.05 was considered statistically significant. Given the small number of deaths (n = 8), prespecified subgroup analyses stratifying by advanced age and higher risk factor burden were deemed underpowered and were therefore not performed to avoid sparse-data bias and overfitting.

## 3. Results

The demographic and clinical characteristics of the 90 patients included in the study are summarized. The mean age of the patients was 74.84 ± 6.65, the median age was 74 (70–79.25), and the age range was 65–94 years. Of the participants, 73.3% were male (n = 66) and 26.7% were female (n = 24). Regarding mode of presentation, 44.4% were NSTEMI (n = 40), 32.2% were STEMI (n = 29), and 23.3% were USAP (n = 21). At the end of one month of follow-up, 91.1% (n = 82) of the patients were alive, while 8.9% (n = 8) had died.

When comorbidities were evaluated, the most common were hypertension (29.0%, n = 69) and diabetes mellitus (28.6%, n = 68). These were followed by COPD (15.1%, n = 36), stroke (9.2%, n = 22), heart failure (6.3%, n = 15), dementia (8.4%, n = 20), and chronic kidney disease (3.4%, n = 8) (Table 1).

The relationship between gender and CIRS-G score Grade 3–4 system number was examined using the Chi-square test. The analysis showed that the distribution of system numbers did not differ between females and males, but the result was close to the threshold of significance (χ^2^ = 8.242, *p* = 0.083). When the distribution was examined, the proportion of males was higher among those with a CIRS-G score Grade 3–4 system number of 1, whereas the proportion of females was higher among those with a value of 3 (Table 2).

There were no significant differences between genders in terms of age, CIRS-G score, frailty score, GRACE score, and EF (all *p* > 0.05). By contrast, the CIRS-G index was significantly higher in females (*p* = 0.008) (Table 3). 

ANOVA analysis by mode of presentation revealed no significant differences between groups for age, CIRS-G score, CIRS-G index, and frailty scores (all *p* > 0.05). By contrast, the GRACE score was significantly higher in the STEMI group (STEMI > NSTEMI, *p* = 0.024; STEMI > USAP, *p* < 0.001). In addition, EF values were significantly higher in the USAP group (USAP > NSTEMI, *p* = 0.017; USAP > STEMI, *p* < 0.001) (Table 4).

In the Mann–Whitney U analysis, at 1-month follow-up, patients who died had significantly higher median values for CIRS-G score (18.5 vs. 14.0; Z = −2.772, *p* = 0.006), CIRS-G index (2.30 vs. 1.90; Z = −1.984, *p* = 0.047), frailty score (6.00 vs. 4.00; Z = −2.674, *p* = 0.008), and GRACE score (183.0 vs. 122.0; Z = −3.901, *p* < 0.001) compared to survivors. Conversely, ejection fraction (EF) was higher in survivors (50.0 vs. 32.5; Z = −3.751, *p* < 0.001) (Table 5).

The Mann–Whitney U analysis revealed no significant differences between smokers and non-smokers regarding CIRS-G score, CIRS-G index, frailty score, or GRACE score (all *p* > 0.05). Conversely, the median ejection fraction (EF) was higher in non-smokers (50.0 vs. 47.5; Z = −2.049, *p* = 0.040) (Table 6).

Spearman correlation analysis showed that CIRS-G score Grade 3–4 system number was positively associated with the CIRS-G score (r = 0.499, *p* < 0.001), CIRS-G index (r = 0.644, *p* < 0.001), and GRACE score (r = 0.408, *p* < 0.001). ICU admission showed a moderate positive correlation with the GRACE score (r = 0.470, *p* < 0.001) and a high negative correlation with EF (r = −0.666, *p* < 0.001). Length of hospital stay was weakly positively correlated with the GRACE score (r = 0.365, *p* < 0.001) and moderately negatively correlated with EF (r = −0.494, *p* < 0.001). In the Pearson correlation analysis, a high positive correlation was found between the CIRS-G score and the frailty score (r = 0.704, *p* < 0.001) and the GRACE score (r = 0.657, *p* < 0.001). In addition, there was a weak negative correlation between the CIRS-G score and EF (r = −0.336, *p* = 0.001). The frailty score was highly positively correlated with the GRACE score (r = 0.702, *p* < 0.001) and weakly negatively correlated with EF (r = −0.306, *p* = 0.003). The GRACE score showed a moderate negative correlation with EF (r = −0.580, *p* < 0.001) (Table 7).

Binary logistic regression was applied to assess the relationship between one-month mortality and the scores. Independent variables were entered simultaneously using the “enter” method: age, GRACE score, CIRS-G score, Clinical Frailty Score, ejection fraction (EF, %), and index diagnosis (NSTEMI, STEMI, USAP; reference category = NSTEMI). Model fit was assessed with the Hosmer–Lemeshow test; explanatory power with Cox–Snell and Nagelkerke R^2^. Classification performance was reported with accuracy, sensitivity, and specificity. The model was overall significant (Omnibus χ^2^(7) = 30.061, *p* < 0.001); explanatory power was moderate-to-high (Cox–Snell R^2^ = 0.284; Nagelkerke R^2^ = 0.629). Fit was good (Hosmer–Lemeshow χ^2^(8) = 6.736, *p* = 0.565). Classification accuracy was 94.4%, with sensitivity 62.5% for death and specificity 97.6% for survivors.

At the variable level, only the GRACE score was independently associated with one-month mortality: OR = 1.081 (95% CI 1.002–1.165), *p* = 0.044. This suggests that every 10-point increase in the GRACE score increases the probability of death by approximately 2.17-fold. Other variables were not statistically significant (*p* > 0.05) (Table 8).

**Figure 1:**
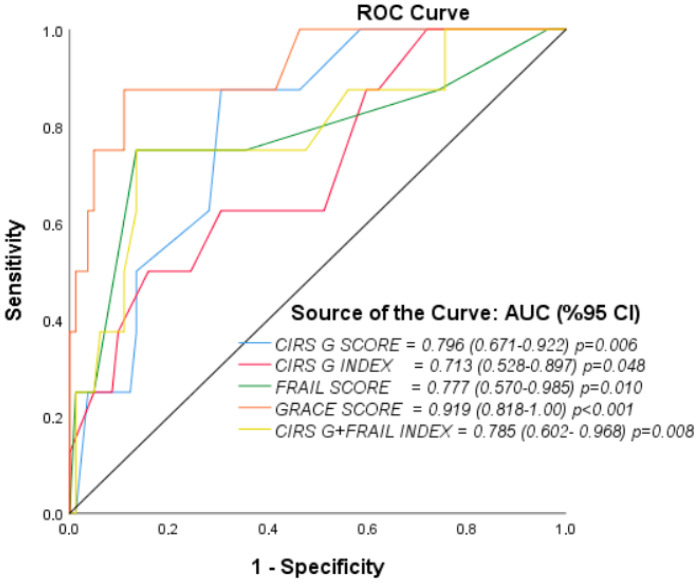
ROC Curves for 1-Month Mortality.

ROC curve analysis was performed to evaluate the discriminative powers in predicting one-month mortality (Figure 1, Table 9). The GRACE score was the parameter that most strongly predicted mortality (AUC = 0.919, 95% CI: 0.818–1.000, *p* < 0.001). According to the Youden index, the optimal cut-off was 124; at this threshold, sensitivity was 100% and specificity 54%. For the CIRS-G score, the AUC was 0.796 (95% CI: 0.671–0.922, *p* = 0.006). The optimal cutoff was 16.5, with sensitivity 88% and specificity 70%. The CIRS-G index showed a relatively lower predictive value (AUC = 0.713, 95% CI: 0.528–0.897, *p* = 0.048). The optimal cut-off was 2.45; sensitivity was 50% and specificity 84%. For the Clinical Frailty Score, the AUC was 0.777 (95% CI: 0.570–0.985, *p* = 0.010). The optimal cut-off was 5.5; sensitivity was 75% and specificity 87%. The combination of the CIRS-G and Clinical Frailty Score provided a predictive value similar to those of the individual scores, with an AUC of 0.785 (95% CI: 0.602–0.968, *p* = 0.008). The optimal cut-off was 0.148; at this threshold, sensitivity was 75% and specificity 87%. Overall, in the comparison of ROC curves, the GRACE score had superior discriminative power over the other parameters, but the combination of the CIRS-G and Clinical Frailty Score also provided clinically meaningful predictive power.

## 4. Discussion

The current retrospective study helps elucidate the prognostic value of comorbidity and frailty scores with GRACE in elderly patients with acute coronary syndrome (ACS). With over 74 years of age, the patients in our cohort correspond to the trend of an increasing age of patients diagnosed with ACS [5]. Similarly to reports from other countries, NSTEMI was the most common form, with STEMI and unstable angina following, which correlates with the epidemiology of Timmis et al. [5] and Neumann et al. [3] who noted the increasing NSTEMI incidence among the older population, in part, due to the better appreciation of this cohort with the introduction of high sensitivity troponin assays. Even with the advances in treatment, our 8.9% one-month mortality rate illustrates the high short term ACS mortality which tends to correlate with the rest of the literature on the subject [1,2].

The results indicate that, in elderly patients diagnosed with ACS, multimorbidity is more prevalent, especially with hypertension and diabetes, as well as other comorbidities such as COPD, stroke, heart failure, dementia, and CKD. The comorbidity profile is similar to that of patients in South Asia and Europe, where Ralapanawa et al. [6] and Bergmark et al. [1] recognized hypertension and diabetes as primary risk factors for the outcomes of ACS. More importantly, the present study adds to the literature that higher comorbidity burdens, as assessed with the CIRS-G, are frailty and adverse outcomes predictors. This is consistent with previous systematic reviews and meta-analyses in the field that demonstrate comorbidity indices are strong predictors of hospital readmission and mortality in elderly patients [7].

In our cohort, frailty was also found to be a determinant of poor outcomes. Those that died within one month had considerably higher frailty scores compared to those that survived, consistent with the results noted by Jamil et al. [8] and Gutierrez et al. [9], who demonstrated that patients with acute myocardial infarction and cardiogenic shock complicated by frailty had markedly worse outcomes and were infrequently treated with invasive procedures. Likewise, Richter et al. [10] and Walker et al. [7] described frailty as a cardiovascular risk factor that transcends age and is underestimated. In this cohort, CIRS-G and GRACE, the comorbidity and hemodynamic risk indices, significantly correlated with the integrated frailty score, which reflects the complex nature of physiological vulnerability. However, frailty, like in the studies by Chung et al. [11] and Kleipool et al. [12], who found a consolidation of predictive domains, did not retain independent predictive power in the multivariable models.

Clinical implications of frailty. In practice, frailty not only identifies patients at higher risk but also often correlates with therapeutic conservatism—frail older adults are less likely to undergo invasive strategies due to perceived procedural risk, which may partly mediate poorer outcomes. Our findings support incorporating structured frailty and comorbidity assessments to inform individualized, risk–benefit–balanced decisions (e.g., invasive vs. conservative management, intensity of secondary prevention, discharge planning), in line with contemporary guidance advocating holistic geriatric evaluation in ACS.

Logistic regression and ROC analyses rigorously validating the GRACE score’s predictive capabilities has also illuminated short-term mortality for other indices, with GRACE continuing to hold the daggers of ACS prognostication with an AUC of 0.919. GRACE has outperformed the comorbidity and frailty indices. Indeed, the CIRS-G AUC of 0.796 and CFS AUC of 0.777 illustrate the clinically relevant discriminative power, especially when considering GRACE’s neglect of functional status and the burden of comorbid disease. Furthermore, Ijaz [13] comments that clinical interventions to target frailty have been shown to improve outcomes in older patients with cardiovascular disease, suggesting that ACS care that incorporates frailty assessment, albeit not parsimonious to the other predictors, could still provide insights to more refined management. There is still, however, the need for progressive rigor that our results demonstrate, advocating the use of GRACE in conjunction with frailty and comorbidity assessments for prognostication to aid more tailored care delivery.

Differences also appeared by sex, where women had markedly higher CIRS-G indices than men. This is supported in part by Mehilli and Presbitero [14], who described how females with ACS tend to be older, face more comorbidity burden, and display more atypical signs and symptoms, making them more complex than their male counterparts in terms of risk stratification and management. The non-significant, yet borderline distributional sex differences in our study in more advanced nonsignificant comorbidity burden also suggest, to a degree, an inequity in biology and/or healthcare access. These findings strengthen the argument to adopt more gender-sensitive strategies in the assessment of frailty and comorbidity in ACS.

Ejection fraction (EF) and outcomes in survivors is also interesting. Survivors had higher EF, and EF was inversely related to both GRACE and frailty scores. This supports the findings of Chang et al. [3], who pointed out that in ACS, some of the most important prognostic factors are the presence of and deficiencies in the heart’s ability to pump blood (i.e., EF) due to the presence of structural and functional changes in the myocardium. Moreover, being admitted to the ICU was associated with having lower EF, further supporting the prognostic significance of left ventricular failure in older patients with ACS. Thus, our study suggests that integrating EF with frailty and comorbidity may improve the assessment of short-term mortality risk.

The status of smoking did not differ with regard to comorbidity or frailty scores but did correlate with lower EF scores. This is consistent with earlier studies showing that smoking left ventricular dysfunction is possible in the absence of smoking-related comorbidity [2,5]. It also underscores the fact that more than geriatric-specific factors are at play.

More broadly, our results add to the discussion on whether frailty and comorbidity should be applied in clinical ACS practice. Although instruments for calculating risks such as GRACE are crucial, the clinical ACS course of elderly patients is also dependent on numerous other factors. As noted by O’Neill and Forman [15], the cardiovascular care of older persons should shift to address frailty and social vulnerability with functional capacity, instead of just the traditional hemodynamic and biomarker risk scores. So too, Won et al. [16] articulated the importance of social support as a psychosocial factor in dealing with frailty and cardiovascular outcomes, highlighting the complex nature of risk in older patients.

In clinical practice, the inclusion of frailty and comorbidity assessments may alter treatment choices. Klein at al. [17], demonstrated how prognostic models designed for the elderly and high-risk patients aid in the decision-making process for invasive versus conservative treatment, particularly in cardiogenic shock advanced stages. Unlike our study, Klein at al. [17] work focuses on treatment suggestions. They did, however, frame frailty and comorbidity as predictors of mortality and strong enough that they could be used to identify patients who may not be appropriate for aggressive treatment and should instead receive supportive or palliative care.

The study also demonstrates the increasing necessity for additional research. Prospective multicenter studies are necessary to confirm the incremental prognostic significance of CIRS-G and CFS in conjunction with GRACE. In addition, the creation of comprehensive geriatric risk models, including the use of machine learning as proposed by Stamate et al., could improve short-term mortality prediction and tailored response formulation, refining the geriatric risk models. Such approaches would alleviate the increasing global burden of elderly patients with ACS and address the gap identified by Richter et al. on the routine use of frailty assessments by cardiologists.

### Limitations

This study has several limitations. First, its single-center and retrospective design may introduce selection and information biases. Second, the relatively small sample size and the limited number of mortality events constrain the robustness of multivariable analyses and reduce statistical power. Finally, potential variability in retrospective frailty assessment could affect measurement consistency. Collectively, these factors may restrict the generalizability of our results and underscore the need for larger, multicenter prospective studies.

## 5. Conclusions

In this study of elderly ACS patients, the GRACE score was confirmed as the strongest independent predictor of one-month mortality, while both CIRS-G and CFS were also significantly associated with adverse outcomes and provided clinically meaningful prognostic value. The combination of comorbidity and frailty scores further enhanced discrimination, suggesting that integrating these geriatric measures with traditional risk models may improve risk stratification and individualized management in this vulnerable population.

## Figures and Tables

**Table 1:** Baseline Characteristics and Cardiovascular Risk Factors.

		Mean ± SD	Median (Q1–Q3)	Min–Max
Age		74.84 ± 6.645	74 (70–79.25)	65–94
		Frequency	Percent (%)	Percent of Cases (%)
Gender	Male	66	73.3	
	Female	24	26.7	
Mode of Presentation	NSTEMİ	40	44.4	
	STEMİ	29	32.2	
	USAP	21	23.3	
1-Month Mortality	Alive	82	91.1	
	Dead	8	8.9	
Total		90	100.0	
Comorbidities	DM	68	28.6%	78.2%
	HT	69	29.0%	79.3%
	COPD	36	15.1%	41.4%
	CVA	22	9.2%	25.3%
	CKD	8	3.4%	9.2%
	HF	15	6.3%	17.2%
	Dementia	20	8.4%	23.0%
Total		238	100.0%	273.6%

**Table 2:** Grade 3–4 Systems by Sex.

				Female		Total			
		n	%	n	%	n	%	*X* ^2^	*p* *
CIRS-G score Grade 3–4 system number	1	34	51.5	7	29.2	41	45.6	8242	0.083
	2	21	31.8	7	29.2	28	31.1		
	3	8	12.1	7	29.2	15	16.7		
	4	3	4.5	2	8.3	5	5.6		
	5	0	0	1	4.2	1	1.1		
	Total	66	100	24	100	90	100		

* Pearson Chi-Square.

**Table 3:** Variables by Sex.

	Male	Female		Mean Difference	95% CI	
	Mean ± SD	Mean ± SD	*p* *		Lower	Upper
Age	74.09 ± 6.22	76.92 ± 7.45	0.074	−2.826	−5.934	0.283
CIRS-G Score	15.24 ± 4.87	15.58 ± 4.10	0.761	−0.341	−2.557	1.875
CIRS-G İndex	1.839 ± 0.476	2.163 ± 0.561	0.008	−0.3231	−0.5599	−0.0863
Frailty Score	4.30 ± 1.31	4.63 ± 1.41	0.316	−0.322	−0.956	0.312
GRACE Score	129.47 ± 26.85	128.46 ± 30.35	0.879	1.011	−12.161	14.183
EF	47.97 ± 9.00	49.63 ± 9.46	0.462	−1.655	−6.159	2.848

* Independent Samples Test.

**Table 4:** Variables by Presentation.

	NSTEMI	STEMI	USAP			Bonferroni		
Variable	Mean ± SD	Mean ± SD	Mean ± SD	F	*p* *	p1	p2	p3
Age	75.10 ± 7.04	75.59 ± 7.03	73.33 ± 5.23	0.749	0.476			
CIRS-G Score	15.73 ± 4.90	16.00 ± 4.42	13.67 ± 4.28	1.818	0.168			
CIRS-G Index	2.05 ± 0.56	1.85 ± 0.47	1.81 ± 0.46	2.085	0.130			
Frailty Score	4.50 ± 1.36	4.66 ± 1.42	3.81 ± 1.03	2.788	0.067			
GRACE Score	129.05 ± 27.01	142.86 ± 27.07	110.62 ± 17.95	9.943	<0.001	0.082	0.024	<0.001
EF	48.85 ± 8.76	43.03 ± 9.53	55.00 ± 5.92	13.615	<0.001	0.012	0.017	<0.001

* ANOVA; p1 = NSTEMİ vs. STEMİ; p2= NSTEMİ vs. USAP; p3= STEMİ vs. USAP.

**Table 5:** Survivors vs. Non-Survivors.

	1 Month Mort.	n	Mean	Std. Deviation	Median	Q1	Q3	Z	*p* *
CIRS-G Score	Alive	82	14.91	4.495	14.00	11.75	18.00	−2.772	0.006
	Dead	8	19.63	4.307	18.50	17.00	24.50		
CIRS-G Index	Alive	82	1.887	0.4966	1.900	1.500	2.125	−1.984	0.047
	Dead	8	2.325	0.5922	2.300	1.800	2.900		
Frailty Score	Alive	82	4.24	1.213	4.00	3.00	5.00	−2.674	0.008
	Dead	8	5.88	1.727	6.00	4.50	7.50		
GRACE Score	Alive	82	124.37	22.318	122.00	108.00	133.00	−3.901	<0.001
	Dead	8	178.75	29.673	183.00	156.25	204.25		
EF	Alive	82	49.68	8.265	50.00	43.75	55.00	−3.751	<0.001
	Dead	8	35.38	7.050	32.50	30.00	43.75		

* Mann–Whitney U.

**Table 6:** Smokers vs. Non-Smokers.

	Smoke	n	Mean	Std. Deviation	Median	Q1	Q3	Z	*p* *
CIRS-G Score	No	48	15.25	4.427	14.50	12.00	18.00	−0.183	0.855
	Yes	42	15.43	4.954	14.50	11.75	18.00		
CIRS-G Index	No	48	1.990	0.5486	1.950	1.600	2.475	−1.225	0.221
	Yes	42	1.852	0.4754	1.850	1.500	2.000		
Frailty Score	No	48	4.56	1.382	4.00	4.00	5.00	−0.851	0.395
	Yes	42	4.19	1.273	4.00	3.00	5.00		
GRACE Score	No	48	127.98	28.893	123.00	108.00	142.00	−0.651	0.515
	Yes	42	130.60	26.447	125.00	113.25	137.00		
EF	No	48	50.29	8.427	50.00	45.00	58.75	−2.049	0.040
	Yes	42	46.26	9.464	47.50	40.00	55.00		

* Mann–Whitney U.

**Table 7:** Correlation Matrix.

		CIRS-G Score	CIRS-G Index	Frailty Score	GRACE Score	EF
CIRS-G score Grade 3–4 system number	Spearman’s rho r	0.499	0.644	0.387	0.408	−0.235
	*p* ^β^	<0.001	<0.001	<0.001	<0.001	0.025
ICU Admission	Spearman’s rho r	0.378	0.304	0.317	0.470	−0.666
	*p* ^β^	<0.001	0.004	0.002	<0.001	<0.001
Length of Hospital Stay	Spearman’s rho r	0.241 *	0.288	0.190	0.365	−0.494
	*p*	0.022	0.006	0.073	<0.001	<0.001
CIRS-G Score	Pearson Correlation r		0.123	0.704	0.657	−0.336
	*p* *		0.249	<0.001	<0.001	0.001
CIRS-G Index	Pearson Correlation r			0.083	0.237	−0.159
	*p* *			0.438	0.025	0.134
Frailty Score	Pearson Correlation r				0.702	−0.306
	*p* *				<0.001	0.003
GRACE Score	Pearson Correlation r					−0.580
	*p* *					<0.001

^β^ Spearman’s rho, * Pearson Correlation.

**Table 8:** Logistic Regression Results.

Variable	B	S.E.	Wald	*p* (Sig.)	OR (Exp(B))	95% CI (Lower)	95% CI (Upper)
Constant	−26.20	7095.53	0.000	1.00	<0.001		
Age	0.083	0.1	0.685	0.408	1.087	0.893	1.322
CIRS-G Score	−0.19	0.264	0.527	0.264	0.827	0.593	1.154
Frailty Score	−0.408	0.408	0.754	0.588	0.665	0.152	2.911
GRACE Score	0.078	0.038	4.063	0.044	1.081	1.002	1.165
Ejection Fraction (EF)	−0.143	0.098	2.148	0.147	0.867	0.715	1.051
NSTEMI_1 _2STEMI			0.0402	0.980			
NSTEMI vs. STEMI	17.258	7095.530	0.000	0.998			
NSTEMI vs. USAP(2)	17.019	7095.530	0.000	0.998			

**Table 9:** ROC Metrics and Cutoffs.

				Asymptotic 95% CI					
	AUC	Std. Error	*p* *	Lower Bound	Upper Bound	Cut-Off	Sensitivity	Specificity	Youden Index
CIRS-G Score	0.796	0.064	0.006	0.671	0.922	16.50	0.88	0.70	0.57
CIRS-G Index	0.713	0.094	0.048	0.528	0.897	2.45	0.50	0.84	0.34
Frailty Score	0.777	0.106	0.010	0.570	0.985	5.50	0.75	0.87	0.62
GRACE Score	0.919	0.052	<0.001	0.818	1.000	124.00	1.00	0.54	0.54
CIRS-G + Frailty Score	0.785	0.094	0.008	0.602	0.968	0.148	0.75	0.87	0.616

* Pearson Correlation.

## Data Availability

The data presented in this study are available on reasonable request from the corresponding author. The data are not publicly available due to privacy and ethical restrictions.

## Abbreviations

The following abbreviations are used in this manuscript:

ACS	Acute Coronary Syndrome
AUC	Area Under the Curve
CKD	Chronic Kidney Disease
COPD	Chronic Obstructive Pulmonary Disease
CFS	Clinical Frailty Score
CIRS-G	Cumulative Illness Rating Scale for Geriatrics
EF	Ejection Fraction
GRACE	Global Registry of Acute Coronary Events
ICU	Intensive Care Unit
NSTEMI	Non-ST-Elevation Myocardial Infarction
ROC	Receiver Operating Characteristic
STEMI	ST-Elevation Myocardial Infarction
USAP	Unstable Angina Pectoris
